# Infection by colombian datura virus induces leaf deformation associated with Indigenous selection of angel’s trumpet (*Brugmansia* spp.)

**DOI:** 10.1007/s13337-025-00939-5

**Published:** 2025-10-14

**Authors:** Sergio A. Hernández-Duarte, Oscar A. Oliveros-Garay, Adriana González, Maria C. Delgado-Niño, Federico Roda

**Affiliations:** 1 Facultad de Ciencias Agrarias, Universidad Nacional de Colombia, Bogotá, 111321 Colombia; 2 Genómica Evolutiva del Metabolismo Especializado (GEME), Max Planck tandem group in collaboration with Universidad Nacional de Colombia, Bogotá, 111321 Colombia

**Keywords:** High-throughput virus screening, Leaf symptoms, Solanaceae, *Brugmansia*, CDV, *Potyvirus trompetae*

## Abstract

Angel’s trumpets (*Brugmansia* spp.) are important medicinal and ornamental plants belonging to the Solanaceae family, domesticated in the Andean region of South America. Indigenous communities in the Colombian Andes have created unique medicinal cultivars exhibiting a variety of unusual shapes in leaves, and specific uses for ritual or medicinal procedures. These cultivars exhibit leaf symptoms typically associated with viral infections, including mosaic, vein chlorosis, and morphological deformities. Colombian datura virus (CDV) is one of the most studied viruses in *Brugmansia*, which is distributed globally and affects even other agriculturally significant hosts. The present study aimed to evaluate the viral infections in Colombian cultivars of the *Brugmansia* genus and the relationship between the presence of viruses and the symptom expression in their leaves. We searched for CDV in the transcriptomes from a set of Solanaceae species and found it mainly in *Brugmansia* medicinal cultivars and wild solanaceous species. Plant material from *B. × candida* with different symptoms was collected and inoculated into plants of representative genera in the Solanaceae family, to evaluate symptom expression. We confirmed the sap of CDV-infected *Brugmansia* ability to cause infections through mechanical inoculation and the CDV presence in the inoculated plants by RT-PCR and Sanger sequencing. Symptoms expressed by these plants included severe leaf deformations in agriculturally important plants such as *Physalis peruviana*, *Solanum lycopersicum*, and for the first time, reported the symptoms of the infection in *S. melongena* and *S. quitoense*.

**Supplementary Information:**

The online version contains supplementary material available at 10.1007/s13337-025-00939-5.

## Introduction

**Fig. 1:**
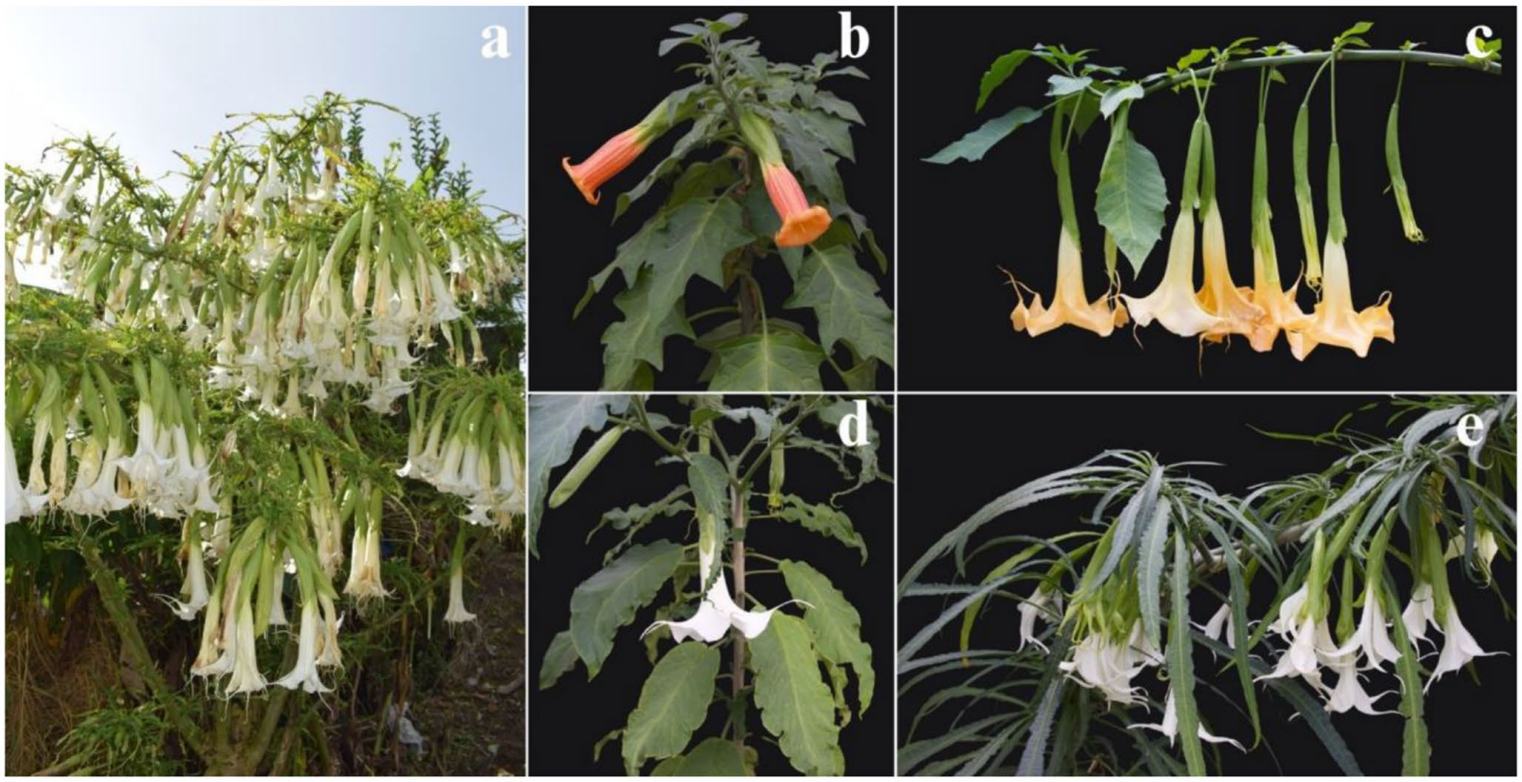
*Brugmansia* is widely recognized for its trumpet-shaped flowers and diverse leaf morphology. **(a)** *B. × candida cv.* ‘Munchiro’, observed in Santiago - Valle del Sibundoy, Putumayo; **(b)** *B. sanguinea*; *B. × candida cultivars*: **(c)** ‘Ocre’, **(d)** ‘Amarón’, **(e)** ‘Culebro’

The *Brugmansia* genus contains some of the most important and widespread ritual and medicinal plants in the Andes. Specifically, Indigenous Peoples in Sibundoy in the department of Putumayo, Colombia, may have selected cultivars with unique morphologies and diverse medicinal uses [1]. *Brugmansia* species are also valued by the pharmaceutical industry as sources of tropane alkaloids [2–4]. In regions such as Sibundoy, this continued use has led to the breeding of stable lineages of *Brugmansia* selected for their psychotropic or medicinal properties [4, 5]. Most of these cultivars from the Sibundoy are interspecific hybrids belonging to the *B. × candida* complex, and exhibit morphologies that cannot be found outside their region of origin. For instance, the ‘Culebro’ cultivar was believed to belong to an undescribed genus by R. E. Schultes, due to its unusually long and narrow leaves and its flowers with free petals (Fig. 1.e); in contrast with members of the genus *Brugmansia* are characterized by petals fused forming a “trumpet” (Fig. 1) [1, 4, 6, 7].

Leaf morphology played a central role in the selection of medicinal cultivars. Leaves are the most used organ in both ritual and medicinal preparations, and their shapes serve as diagnostic features for cultivar identification by traditional healers. This resulted in an unusual diversification of leaves in the Sibundoy region. Cultivars such as ‘Buyés’, ‘Dientes’, and ‘Ocre’ are characterized by ovate to elliptical leaves, which are common in the *Brugmansia* genus. However, in ‘Dientes’, younger leaves occasionally exhibit distinctive deformities in shape and serrated margins. Furthermore, cultivars such as ‘Amarón’, ‘Munchiro’, and ‘Quinde’ have asymmetrical leaf shapes, often exhibiting deformations and asymmetries of the leaf blades that can resemble the effect of insect herbivory [1].

Two non-exclusive hypotheses have been proposed to explain the diversity of leaf shapes in indigenous medicinal cultivars, namely (1) heritable genetic mutations and (2) viral infection. The first, more widely accepted hypothesis suggests that traditional selection of genotypes carries beneficial mutations [1, 4]; virus-free progeny from *B. x candida* cv. ‘Quinde’ exhibited the mutant leaf shape, suggesting a genetic basis rather than a viral origin [1]. Second, the viral disease hypothesis has been overlooked, anecdotal accounts suggest that viral infection is involved in the induction of leaf malformations in at least some medicinal cultivars. Leaf deformities are environmentally dependent and variable within the same plant, often appearing and disappearing through time and development [7]. Importantly, sap from symptomatic plants can induce deformities in asymptomatic individuals, indicating a transmissible agent [1].

Finally, at least three viruses of genus *Potyvirus* have been detected in *Brugmansia* spp: colombian datura virus (CDV), brugmansia suaveolens mottle virus (BsMoV), and brugmansia mosaic virus (BruMV) [8–12]. Vegetative propagation *via* clonal cuttings of *Brugmansia* may facilitate the spread of these viruses [11]. CDV is the most studied potyvirus in *Brugmansia* spp., which induces characteristic foliar symptoms: mosaic, rugosity, and chlorotic spots that initially appear at the tip of the leaf and then spread across the blade, along with vein yellowing and alterations in leaf development, including severe deformations [7]. Importantly, genetic diversity studies have found that CDV likely originated in the Putumayo and displays very low variability, which has been linked to anthropogenic selection of CDV isolates infecting Colombian native cultivars [10]. CDV was recently dispersed worldwide, affecting a range of agricultural hosts such as *Solanum lycopersicum*, *Nicotiana tabacum*, *Cucumis sativus*, *S. muricatum*, *Physalis alkekengi*, and *P. peruviana* [10, 12–15]. Objectives of this study were: (1) to detect CDV infection in native cultivars of *B. × candida* from the departments of Nariño and Putumayo in Colombia. These plants were propagated from cuttings and seeds and now constitute an *ex-situ* collection at Universidad Nacional de Colombia, Bogotá; (2) to inoculate CDV into plants of other Solanaceae genera to identify species that express symptoms of leaf deformation like those exhibited in some native cultivars of *B. × candida*. Among the evaluated Solanaceae species are several of agricultural importance.

## Materials and methods

### Plant material

The plants used in this study were part of a living collection maintained in the greenhouse of the Max Planck Tandem group “Genomica Evolutiva del Metabolismo Especializado” (GEME) at the Universidad Nacional de Colombia. Medicinal cultivars of *Brugmansia* were collected in the departments of Nariño and Putumayo in Colombia and propagated from cuttings and seeds in the greenhouse. All samples were collected legally under the “Permiso Marco de Recolección de Especímenes de Especies Silvestres de la Diversidad Biológica con Fines de Investigación Científica No Comercial”, granted to the Universidad Nacional de Colombia by the Ministerio de Medio Ambiente (Resolución 0255 del 12 de marzo de 2014). The collection is maintained under controlled conditions at a constant temperature of 32 °C and 75% relative humidity. In this study, we selected the following medicinal cultivars of *B. × candida*: ‘Buyés’, ‘Ocre’, ‘Quinde’, ‘Amarón’, ‘Munchiro’, ‘Culebro’ and ‘Dientes’. Leaves and roots from these plants were previously characterized using RNA sequencing (RNAseq).

**Virus detection using transcriptomic data.** We first searched for viral sequences in the transcriptomic data of 130 wild and domesticated species, representing the major clades in the Solanaceae family in Colombia. These plants are part of a living collection maintained by the GEME group in the greenhouse of the Universidad Nacional de Colombia, Bogotá. The plants in this collection were derived from seeds and cuttings collected from the field. We previously performed RNA seq on leaves and roots, and the data were deposited in GenBank under accession code PRJNA1070281. Reads were cleaned with Trimmomatic [16], and *de novo* transcriptomes from various species were constructed with the Trinity software [17].

We searched for viral sequences in the transcriptomes of Solanaceae species by conducting a BLASTn search [18], against sequences found in the global database of plant viruses (DPVweb) [19]. We retrieved transcriptome hits with a minimum of 90% identity to viral sequences. The results were subjected to a filtering process to select sequences with lengths exceeding the average of all identified sequences (> 3000 nt). This step facilitated the necessary overlap for the subsequent alignment. The sequences were aligned using the ‘Codoncode’ version 2.0.1 (CodonCode Co.) and ‘MUSCLE’ [20] software to edit alignments and identify polymorphisms between sequences.

### Evolutionary relationships

To analyze evolutionary relationships between CDV sequences, phylogenetic trees were constructed using the MEGA 11 software [21], employing the Maximum Likelihood and Neighbor- Joining methods with a Bootstrap test of 1000 replicates. The analysis was based on partial gene CP sequences of CDV. The sequences used in the phylogenetic analysis, along with their corresponding NCBI/GenBank accession numbers, are listed in Table 1.

**Table 1:** Sequences of CP gene of CDV used in phylogenetic analysis from solanaceous Colombian collection and GenBank

Potyvirus	Origin	GenBank
CDV isolate from *B. × candida* ‘Biangan’	COL	PV781182
CDV isolate from *B. × candida* ‘Buyés’	COL	PV781179
CDV isolate from *B. × candida* ‘Culebro’	COL	PV781184
CDV isolate from *B. × candida* ‘Dientes’	COL	PV781177
CDV isolate from *B. × candida* ‘Munchiro’	COL	PV781178
CDV isolate from *B. × candida* ‘Ocre’	COL	PV781180
CDV isolate from *B. × candida* ‘Quinde’	COL	PV781181
CDV isolate from *B. insignis* ‘Andaqui’	COL	PV781183
CDV isolate from *Brugmansia suaveolens*	KOR	MW075268.1
CDV isolate from *Brugmansia suaveolens*	KOR	OL999301.1
CDV isolate from *Nicotiana benthamiana*	TW	LC771070.1
CDV isolate from *Nicotiana tabacum*	GER	OQ847405.1
CDV isolate from *Nicotiana tabacum*	UK	JQ801448.1
CDV isolate from *Nicotiana tabacum*	USA	NC_020072.1
Celery latent virus	ITA	MH932227.1
Pokeweed mosaic virus	USA	JQ609095.1
Potato virus A	HU	AJ296311.1
Potato yellow blotch virus	UK	JX294310.1
Sunflower mild mosaic virus	ARG	JQ350738.1
Tamarillo leaf malformation virus	COL	KM523548.1
Tobacco etch virus		M11458.1
Tobacco vein mottling virus	USA	X04083.1

Abbreviations: ARG: Argentina; COL: Colombia; TW: Taiwan; HUN: Hungary; ITA: Italy; KOR: Korea; UK: United Kingdom; USA: United States

### Inoculation of plants

Mechanical inoculation tests were performed using sap extracted from symptomatic leaves (deformed and asymmetric or with mosaic, yellowing) of *B. × candida* cultivars from the GEME group collection under greenhouse conditions. A total of 0.5 g of symptomatic plant material in 5 mL of phosphate buffer (10 mM, pH 7.0), following the PM 7/153 (1) protocol [22]. Inoculation was performed on test plants of the following species: *S. lycopersicum*, *S. melongena*, *S. quitoense*,* P. peruviana*, *B. sanguínea*, *N. tabacum*, *P. hybrida* and *N. glutinosa*. Controlled inoculation assays were not performed on *B. × candida* cultivars, as all specimens in the collection exhibited symptoms consistent with viral disease. Expressed symptoms were evaluated between 7 and 21 dpi. Infections were carried out on mature leaves, 40 days after emergence, with 3–6 repetitions for each *Brugmansia* cultivar selected as the inoculum source. Negative control consisted of plants without sap inoculation.

### DsRNA and RNA extraction

Double-stranded RNA (dsRNA) was extracted from the selected *Brugmansia* samples. To achieve this, 15 g of infected tissue was macerated and pulverized in liquid nitrogen. The extraction protocol used corresponds to that previously reported by [23, 24]. Total RNA was extracted from 100 mg of tissue using the TRIzol^®^ reagent (Invitrogen) according to the manufacturer’s standard method. The resulting RNA pellet was then resuspended in 100 µL of DEPC-treated water. RNA was subjected to electrophoresis at 80 V in a 1.5% agarose gel, using a 1000 kb ladder. Staining was carried out with ethidium bromide, and visualization was performed on Bio-Rad Laboratories’ ChemiDoc MP imaging system.

### Molecular detection

A detection assay was conducted using reverse transcription (RT) and polymerase chain reaction (PCR) with degenerate primers targeting the potyvirus CI and NIb region [25, 26], and specific primers for CDV targeting NIb/CP region [10] (see Table 2). Reverse transcription of total RNA and dsRNA was performed using the High-Capacity cDNA Reverse Transcription Kit by Applied Biosystems™. For primers NIb2f and NIb3R (Table 2), PCR was performed for 35 cycles: 95 °C for 45s, 45 °C for 45s, 72 °C for 45s, and a final extension at 72 °C for 5 min. For primers CIfor and CIrev (Table 2), PCR was conducted for 40 cycles: 94 °C for 30s, 40 °C for 30s, 72 °C for 1 min, and a final extension at 72 °C for 5 min. For CDVv and CDVvc (Table 2), PCR was run for 30 cycles: 94 °C for 30s, 55 °C for 45s, 72 °C for 1 min, and a final extension at 72 °C for 5 min. The positive control corresponded to RNA extracted from plants with viral RNA detected through BLAST analysis of RNA-seq. The negative control consisted of indicator plants grown from seeds known to be free of viral agents, and the blank control used specific primers without a sample. The products obtained from each amplification were analyzed under the same electrophoresis conditions as previously described.

**Table 2:** Primers, oligonucleotide sequences, expected PCR fragments sizes, and references

Name	Sequence	Size (pb)	Degenerate/ Specific	References
NIB2F	GTITGYGTIGAYGAYTTYAAYAA	350	Degenerate	[26]
NIB3R	TCIACIACIGTIGAIGGYTGNCC			
CIfor	GGIVVIGTIGGIWSIGGIAARTCIAC	700	Degenerate	[25]
CIrev	ACICCRTTYTCDATDATRTTIGTIGC			
CDVv	GGGAGAGCTCCTTACCTAGC	511	Specific	[10]
CDVvc	CCATGTATGTTTGGTGACGTACC			

RNA extracted from *Brugmansia* cultivars and inoculated solanaceous plants showing symptoms associated with viral infection was subjected to RT-PCR amplification using specific CDV primers. The purified PCR products were sequenced using Sanger technology, and the resulting sequences were aligned using BLAST for further analysis.

## Results

### Detection of CDV through transcriptome analysis of Solanaceae family plants, including the *Brugmansia* genus

The leaf transcriptomes of Solanaceae 135 species from 35 genera collected throughout Colombia were evaluated using BLAST to search for plant virus sequences. We detected CDV sequences in all medicinal *Brugmansia* cultivars (‘Ocre’, ‘Munchiro’, ‘Buyés’, ‘Dientes’, ‘Culebro’, ‘Biangán’, ‘Quinde’, ‘Andaqui,’ ‘Amarón’) as well as *B. suaveolens*,* B. sanquinea*. We also detected CDV in other wild and cultivated solanaceous species, including *Datura wrightii*, *Atropa belladonna*, *Browallia americana*, *Lycianthes amatitlanensis*, *Solanum catilliflorum*,* S. sect. Cyphomandra*,* Jaltomata* sp. and *S. tuberosum* (Online Resource 1). The nucleotide identity percentages for CDV sequences ranging from 97.2 to 100%; the only low value was *S. tuberosum*, where the identity was 80.8%. Additionally, transcriptomic short contigs with 80%-85% nucleotide identity to tobacco vein clearing virus (TVCV) were detected in most solanaceous plants from the collection, including cultivars of *Brugmansia* sp., as determined by BLASTn analysis (Online Resource 2).

**Fig. 2:**
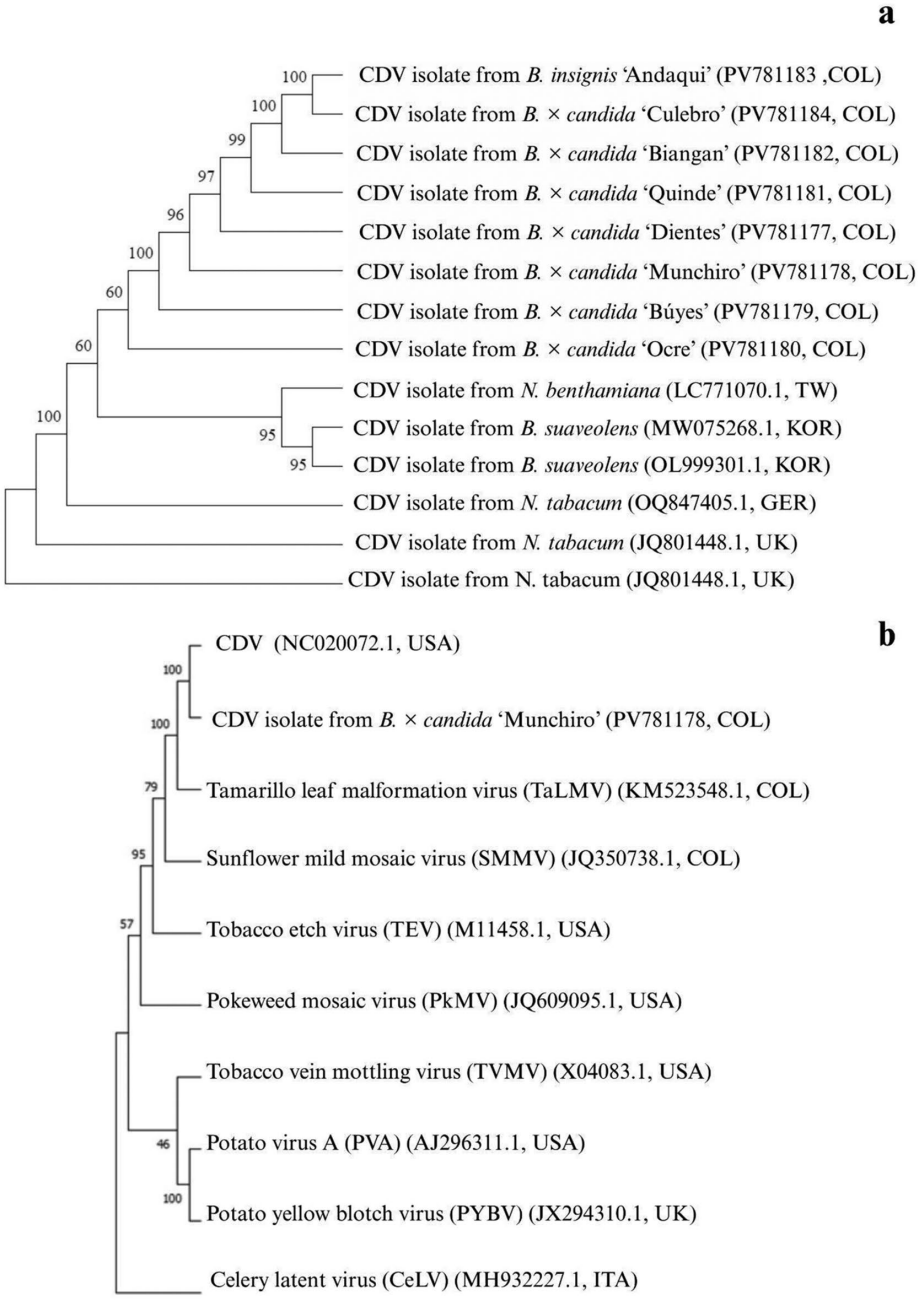
**(a).** Neighbor-joining phylogenetic tree based on deduced polypeptide sequences of CDV CP gene and other representative partial isolates from different regions. Bootstrap analysis was performed with 1,000 replicates, using accession NC_020072 as the outgroup. (**b).** Neighbor-joining phylogenetic tree based on partial CP sequences of Colombian CDV isolates, the reference genome (NC_020072), and other potyviruses. Bootstrap analysis was realized with 1,000 replicates. Celery latent viru*s* (MH932227.1), a virus of genus *Celavirus* within the family *Potyviridae*, served as the outgroup

To identify the closest relatives of the CDV isolates detected in our Solanaceae collection, we compared partial sequences longer than 1000 bp with reference CDV genomes from different countries. The alignments of putative sequences of gene CP in native cultivars matched representative global isolates (Fig. 2a). CP gene sequences from the reference genome (accession NC_020072 NCBI) and *B. × candida* cv. ‘Munchiro’ were also compared with other potyvirus sequences (Fig. 2b). A closer evolutionary relationship was found between CDV and tamarillo leaf malformation virus TLMV (accession KM523548.1) a greater evolutionary divergence was observed with celerity latent virus (accession MH932227.1), the most distant genus within the family *Potyviridae*.

### Symptoms associated with viral infection in *B. × Candida* cultivars

**Fig. 3:**
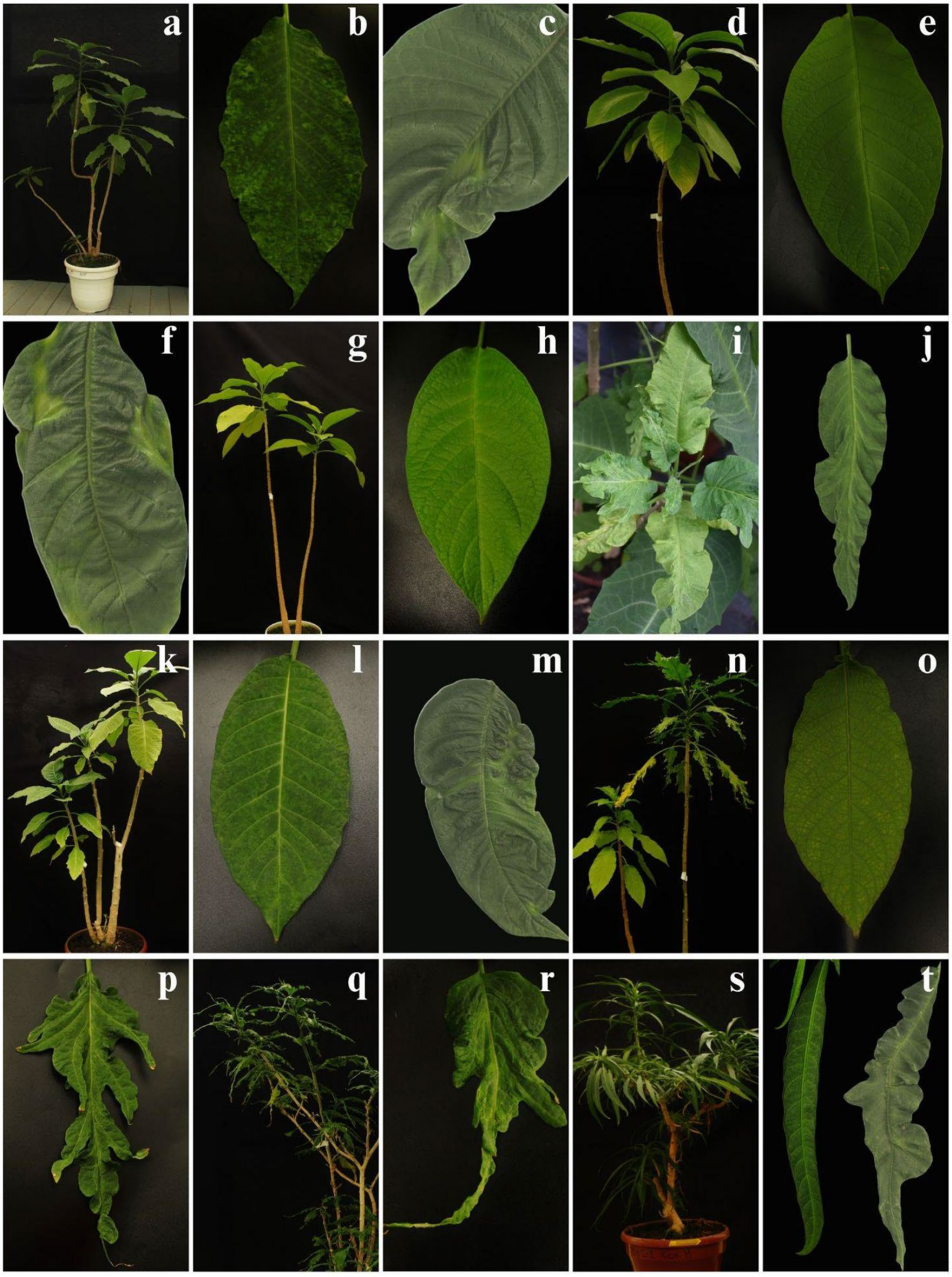
Symptoms observed in the leaves of *B. × candida* cultivars infected with CDV. **OL**: ovate leaf; **DL**: deformed leaf; **MP**: mosaic pattern; **VY**: vein yellowing; **SL**: slender leaf; **W**: without symptoms; **AC**: apical curling. **(a-c)** ‘Dientes’: MP, VY, DL. **(d-f)** ‘Ocre’: W, MP, DL. **(g-j)** ‘Amarón’: Variable leaf development, including DL, interveinal chlorosis, and AC; OL. **(k-m)** ‘Buyés’: VY, MP, DL. **(n-p)** ‘Quinde’: Heterogeneous leaf development with plants showing W, or DL, VY, AC in symptomatic branches. **(q-r)** ‘Munchiro’: VY, AC, and DL. **(s-t)** ‘Culebro’: SL and irregular leaf blade, MP, VY, and AC. GEME collection of *Brugmansia* species grown under greenhouse conditions at the Universidad Nacional de Colombia, Sede Bogotá

The plants from the *Brugmansia* native cultivars collection were evaluated for expression leaf symptoms (Fig. 3). We observed several cultivars exhibiting mosaic patterns, including ‘Dientes’, ‘Ocre’, Amarón, and ‘Buyés’ (Fig. 3b, f, j, m). Additionally, some cultivars showed rough and asymmetric leaves, with this symptom being more prominent in ‘Munchiro’, ‘Quinde’, and ‘Amarón’ (Fig. 3j, p, r). Cultivars such as ‘Ocre’, ‘Buyés’, and ‘Dientes’, displayed most leaves with a regular ovate shape (3h, e, l); however, some of these cultivars can exhibit leaf malformation in young shoots (Fig. 3c, f, m). In other cultivars, like ‘Amaron’ and ‘Quinde’ some leaves were ovate while others were asymmetrical (Fig. 3h, o). ‘Culebro’ presented long leaves, occasionally irregular in shape (Fig. 3t). Finally, cultivars ‘Culebro’, ‘Munchiro’, and ‘Quinde’ can exhibit apical curling (Fig. 3p, r, t).

### Solanaceae plants inoculated with Sap from cultivars of *Brugmansia × Candida*

**Fig. 4:**
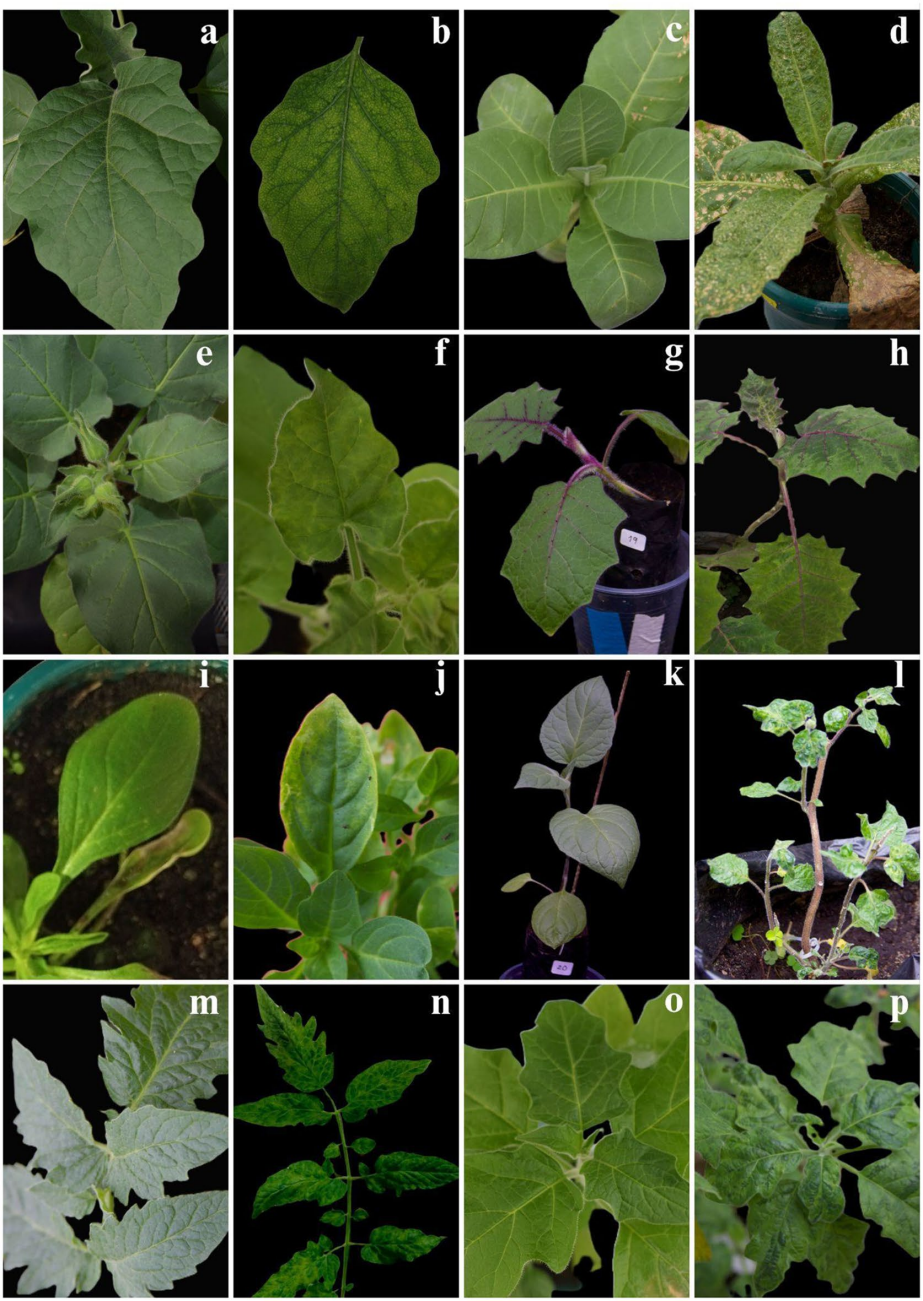
Symptoms observed in mechanically inoculated plants using sap extracted from *B. × candida* cv. ‘Munchiro’, which showed systemic symptoms and tested positive for CDV by RT-PCR and sequencing. Mock-inoculated (**a**, **c**, **e**,** g**,** i**,** k**,** m**,** o)** and sap-inoculated (**b**,** d**,** f**,** h**,** j**,** l**,** n**,** p)** plants. **(a**,** b)** *S. melongena*; (**c**,** d)** *N. tabacum*; **(e**,** f)** *N. glutinosa;* **(g**,**h**) *S. quitoense*; **(i**,**j**) *Petunia* × *hybrida*; **(k**,** l)** *P. peruviana*; **(m**,**n**) *S. lycopersicum*; **(o**,**p**) *B. sanguinea*

Since RNA-seq results indicated the presence of CDV, we performed mechanical inoculations using sap extracted from several cultivars of *B. × candida* medicinal cultivars onto different solanaceous species. Typical symptoms including mosaic, leaf folding and/or necrosis were observed (Table 3). In a subsequent assay, sap from the cultivar cv. ‘Munchiro’, which exhibited leaf deformities, was used as an inoculum source to evaluate whether CDV and associated symptoms could be transmitted to Solanaceae species of agricultural interest, namely *S. melongena*, *N. tabacum*, *S. lycopersicum*,* S. quitoense*, and *P. peruviana*. Symptom expression in the test Solanaceae plants inoculated is described below. *S. melongena* exhibits interveinal chlorosis on the leaf blades progressing to necrotic spots in later stages of infection (Fig. 4. a, b). In *N. tabacum*, interveinal chlorosis begins at the base of the leaf blade and gradually progresses to a generalized mosaic and curling of the leaves at 20 days post inoculation (dpi); by 40 dpi chlorotic spots develop into widespread necrosis (Fig. 4. c, d). In *N. glutinosa*, chlorotic spots extend from the base to the tip of the leaves, progressing into chlorotic mottling, with curling and deformities are also observed (Fig. 4. e, f). *S. quitoense* shows a generalized mosaic and leaf deformation (Fig. 4. g, h). In *P. hybrida*, chlorotic spots appear along the edges, interveinal yellowing, and mosaic pattern (Fig. 4. i, j). *P. peruviana* displays twisting of leaves and branches, a generalized mosaic pattern, and severe deformation of the leaf blade (Fig. 4. k, l). *S. lycopersicum* presents chlorotic spots, vein chlorosis, mosaic patterns, and twisting of branches (Fig. 4. m, n). Finally, we inoculated *B*. *sanguinea* because it was possible to access CDV-free plants, to induce symptom expression after experimental inoculation; these inoculated plants showed a mosaic pattern 20 dpi, and leaf blade folding and deformation in younger leaves (Fig. 4. o, p).

**Table 3:** Symptoms of viral infection in solanaceaus plants inoculated with Sap from *B. × Candida* cultivars

Inoculum source- B. × candida cultivars	Solanaceae plants^1^							
	NT	NG	SM	PP	PH	SL	BS	SQ
‘Dientes’	5/6	3/3	2/4	5/6	3/4	4/6	3/3	-
‘Ocre’	4/6	3/3	1/4	3/6	-	-	-	-
‘Amarón’	5/6	3/3	3/4	5/6	-	-	-	1/6
‘Buyés 1’	4/6	3/3	2/4	2/6	2/4	4/6	2/3	-
‘Buyés 2’	4/6	3/3	3/4	4/6	2/4	5/6	3/3	-
‘Quinde’ (symptomatic)	5/6	3/3	4/4	5/6	-	-	-	-
‘Quinde’ (non-symptomatic)	2/6	2/3	3/4	3/6	-	-	-	-
‘Munchiro’	3/6	3/3	2/4	5/6	3/4	5/6	3/3	1/6
‘Culebro’	4/6	3/3	3/4	4/6	-	-	-	-

1 Abbreviations: NT, N. tabacum; NG, N. glutinosa; SM, S. melongena; PP, P. peruviana; PH, P. hybrida; SL, S. lycopersicum; BS, B. sanguinea; SQ, S. quitoense; –, inoculation non developed

RT-PCR and sequencing were employed to confirm the presence of CDV in *Brugmansia* cultivars and inoculated plants. We initially amplified RT-PCR fragments using degenerate potyvirus primers designed to target conserved regions within the CI coding sequence (Table 2). Reactions with primers NIb2F/NIb3R and CIFor/CIRev yielded amplicons of ~ 350 bp and ~ 700 bp respectively (Online Resource 3a and b). Instead, specific primers CDVv and CDVvc targeting the partial NIb/CP region, which consistently produced amplicons of 511 nucleotides across all symptomatic *B. × candida* cultivars and inoculated Solanaceae species (Online Resource 3c). BLAST analysis of partial, specific PCR products from CP gene confirmed the identity of this virus with values above 99% (Online Resource 4). Amplification was specific to all *B. × candida* cultivars, including those transmitted to test Solanaceae plants. Nucleotide sequences of 17 isolates of CDV from this study were deposited in GenBank with accession numbers PQ869650 to PQ869666.

## Discussion

We evaluated the evolutionary relationships of CDV isolates by two experimental strategies: (i) Transcriptome analysis of several Solanaceae species collected in Colombia revealed the presence of CDV in *B. suaveolens*,* B. insignis*,* B. sanguinea* and several *B. × candida* medicinal cultivars from the Putumayo region, as well as wild and cultivated solanaceous species from other genera. Phylogenetic analysis showed a close evolutionary relationship and low genetic variation between the CDV isolates in this study and those reported in studies from different geographic regions. This pattern has been linked to human selection of viral isolates infecting *Brugmansia* native cultivars from the Andes [10]. (ii) The analysis of **s**equences of partial regions of the NIb and CP genes obtained using degenerate primers revealed identity levels of less than 75% when compared to other known potyviruses. Nucleotide comparisons revealed 71% nucleotide identity of tamarillo leaf malformation virus (TLMV) with CDV isolates from *B. × candida*, which has been previously reported [27, 28]. Phylogenetic analysis indicated that TLMV is the closest virus relative to CDV, followed by five other potyviruses: sunflower mild mosaic virus (SMMV), tobacco etch virus (TEV), pokeweed mosaic virus (PkMV), potato virus A (PVA), and tobacco vein mottling virus (TVMV). These observations align with previous reports [29–31]. Although some short reads related to TVCV were detected in the *Brugmansia* transcriptome through BLAST-based analysis, this pararetrovirus has been identified only in *N. edwarsonii* and it was not possible to infect other Solanaceae species by mechanical inoculation [32]. The presence of pararetrovirus-related sequences has been previously identified in several Solanaceae [33, 34 ]. However, the TVCV-like sequences identified in our dataset by BLASTn showed a low identity with this pararetrovirus. We propose that full-length sequences of this putative pararetrovirus, as identified in the transcriptome of *Brugmansia* and other Solanaceae genera, should be obtained to clarify its potential relationship to TVCV.

The plants inoculated with sap of CDV-infected *Brugmansia* exhibited a variety of symptoms associated with viral infection: mosaic patterns, vein chlorosis, chlorotic spots and leaf malformations. Other potyviruses can induce symptoms of leaf blade deformation in their plant hosts. For instance, papaya ringspot virus (PRSV) induces severe leaf blade distortion in *Carica papaya* L [35]. Additionally, symptoms generated by TLMV in *S. betaceum* include mosaics and severe leaf blade deformations [27]. Our results show that all evaluated medicinal cultivars are infected with CDV, independently of their leaf morphology, but CDV inoculation can induce leaf deformations. These findings suggest that CDV infection may have played a role in the domestication of *Brugmansia* medicinal cultivars. However, CDV infection may not be the sole factor associated with the induction of leaf deformities. Further investigation is needed to determine why CDV induces leaf deformities in some plants.

Natural hosts of CDV, primarily within the Solanaceae family, have been reported. In *S. lycopersicum*, CDV was detected in cultivated plants exhibiting viral symptoms [14]. The presence and expression of symptoms were also observed in *P. peruviana*, *S. muricatum*, and *Mandragora officinarum* L [13, 36]. Additionally, while CDV infection in *S. tuberosum* has been suggested under controlled inoculation, natural infections have not yet been documented [37, 38]. We demonstrated the expression of symptoms in solanaceous plants infected experimentally which expressed similar symptoms to those previously reported in *N. tabacum*, *N. glutinosa*, *P. peruviana*, *P. hybrida*, *S. lycopersicum* [10, 13, 14, 39, 40]. Our study confirms the variability in symptom expression induced by CDV in *N. tabacum*, including mosaic and severe necrosis [40]. Regarding leaf deformation induced by the sap of CDV-infected *Brugmansia*, *S. quitoense*, *B. sanguinea* and *P. peruviana* were the hosts with the greatest leaf deformities. Notably, CDV also induces systemic leaf deformities in several solanaceous hosts, including *Datura medel*,* P. × hybrida*,* S. lycopersicum*, *Browallia demissa*, *N. glutinosa*, *S. nigrum*, and *S. scabrum*, reinforcing its role in altering plant development [10, 13].

Given the variability in symptom expression, we propose possible mechanisms underlying these morphological changes, particularly those related to auxin signaling that plays a crucial role in leaf morphogenesis and vascular development, regulated by Auxin Response Factors (ARFs) [41, 42]. Developmental anomalies linked to viral suppressors of RNA silencing (VSRs) have been reported in potyvirus-infected plants, where ARF8 misregulation underlies morphological abnormalities in transgenic *Arabidopsis thaliana* expressing VSRs [43]. Similarly, in turnip mosaic virus (TuMV) infections, HC-Pro VSR disrupts miR167-mediated auxin regulation, leading to altered leaf development [44]. Other potyviruses, including tobacco etch virus (TEV) and potato virus Y (PVY), have been shown to induce differential miRNA expression in host plants, affecting auxin-related pathways and contributing to developmental abnormalities in *S. lycopersicum* and *C. papaya* [45, 46]. Whether similar mechanisms contribute to symptom development in *B. × candida* and other cultivated Solanaceae remains an open question.

Our study suggests that CDV can induce leaf deformation in plants of the *Brugmansia* genus, which may have influenced the selection of cultivars by Indigenous communities based on leaf morphological traits and medicinal uses. We propose that virus-induced traits could have contributed to the selection of specific phenotypes during early domestication, potentially favored in ritual or medicinal contexts. This hypothesis suggests a possible role of CDV in shaping the evolutionary trajectory of *B. × candida* cultivars under human influence. These findings emphasize the need to investigate the mechanisms underlying leaf deformation in CDV-infected solanaceous plants, particularly in *B. × candida* cultivars with distorted leaf morphology.

## Electronic supplementary material

Below is the link to the electronic supplementary material.

Supplementary Material 1
