# Supplementary material for: Infection by colombian datura virus induces leaf deformation associated with Indigenous selection of angel’s trumpet (Brugmansia spp.)
Source: Virusdisease. 2025 Oct 14;36(3):506–17. doi: 10.1007/s13337-025-00939-5 (PMC12634988; doi:10.1007/s13337-025-00939-5)
Supplement: Supplementary file 1 — Supplementary Material 1 [file 13337_2025_939_MOESM1_ESM.docx]

**Online Resources**

**Online Resource 1.** BLAST analysis results of CDV sequences in Solanaceae collection

| **Cultivar** | **Pident %** | **evalue** | **Bit score** |
| --- | --- | --- | --- |
| *Atropa belladonna* | 97.98 | 1.25e-120 | 431 |
| *Browallia americana* | 98.38 | 9.63e-122 | 435 |
| *Brugmansia ‘Amarón’* | 98.71 | 3.27e-157 | 553 |
| *Brugmansia ‘Buyés’* | 98.60 | 0 | 17023 |
| *Brugmansia ‘Culebro’* | 97.33 | 0 | 16310 |
| *Brugmansia ‘Dientes’* | 97.31 | 0 | 16299 |
| *Brugmansia ‘Munchiro’* | 98.17 | 0 | 16790 |
| *Brugmansia ‘Ocre’* | 98.88 | 2e-178 | 17294 |
| *Brugmansia ‘Quinde’* | 97.39 | 0 | 11365 |
| *Brugmansia Andaqui* | 97.19 | 0 | 9856 |
| *Brugmansia Biangán* | 97.40 | 0 | 13208 |
| *Brugmansia sanguinea* | 98.37 | 0 | 645 |
| *Brugmansia suaveolens* | 99.10 | 0 | 1002 |
| *Datura wrightii* | 98.19 | 0 | 8403 |
| *Ipomoea purpurea* | 100 | 6.35e-108 | 388 |
| *Lycianthes amatitlanensis* | 97.50 | 1.62e-114 | 411 |
| *Solanum catilliflorum* | 98.51 | 4.13E-129 | 473 |
| *Solanum sect. Cyphomandra* | 99.45 | 0 | 658 |
| *Solanum tuberosum* | 80.80 | 2.51E-101 | 381 |

**Online Resource 2.** BLAST analysis results of TVCV sequences in Solanaceae collection

| **Sample** | **Virus** | **Pident %** | **length** | **evalue** | **bitscore** |
| --- | --- | --- | --- | --- | --- |
| *Brugmansia 'Amarón'* | TVCV | 82.73 | 2715 | 0 | 2337 |
| *Brugmansia 'Andaqui'* | TVCV | 84.54 | 595 | 5.6E-162 | 577 |
| *Brugmansia arborea* | TVCV | 81.52 | 1542 | 0 | 1249 |
| *Brugmansia aurea* | TVCV | 81.66 | 2061 | 0 | 1677 |
| *Brugmansia 'Biangán'* | TVCV | 81.20 | 649 | 1.6E-141 | 508 |
| *Brugmansia 'Buyés'* | TVCV | 84.43 | 1381 | 0 | 1345 |
| *Brugmansia 'Culebra'* | TVCV | 85.97 | 1119 | 0 | 1188 |
| *Brugmansia insignis* | TVCV | 81.86 | 1858 | 0 | 1517 |
| *Brugmansia 'Munchiro'* | TVCV | 82.86 | 875 | 0 | 769 |
| *Brugmansia 'Ocre'* | TVCV | 80.46 | 2564 | 0 | 1877 |
| *Brugmansia 'Quinde'* | TVCV | 85.57 | 1448 | 0 | 1495 |
| *Brugmansia sanguinea* | TVCV | 84.20 | 1209 | 0 | 1164 |
| *Brugmansia sanguinea x vulcanicola* | TVCV | 82.94 | 2497 | 0 | 2220 |
| *Brugmansia suaveolens* | TVCV | 80.59 | 2607 | 0 | 1960 |
| *Brugmansia versicolor* | TVCV | 81.33 | 2137 | 0 | 1685 |
| *Brugmansia vulcanicola* | TVCV | 84.80 | 1539 | 0 | 1535 |
| *Cuatresia sp. nov* | TVCV | 83.47 | 1518 | 0 | 1376 |
| *Datura wrightii* | TVCV | 82.66 | 1407 | 0 | 1236 |
| *Doselia lopezii* | TVCV | 83.68 | 582 | 5.7E-149 | 534 |
| *Iochroma arborescens* | TVCV | 83.06 | 1334 | 0 | 1177 |
| *Jaltomata procumbens* | TVCV | 84.87 | 1117 | 0 | 1120 |
| *Juanulloa ochracea* | TVCV | 83.75 | 966 | 0 | 907 |
| *Lycianthes sp.* | TVCV | 80.43 | 1553 | 0 | 1125 |
| *Markea pilosa* | TVCV | 82.04 | 1253 | 0 | 1016 |
| *Markea sp nov* | TVCV | 81.89 | 2214 | 0 | 1807 |
| *Physalis peruviana* | TVCV | 85.35 | 348 | 1.11E-95 | 355 |
| *Schultesianthus coriaceus* | TVCV | 83.74 | 1501 | 0 | 1393 |
| *Solanum crinitipes* | TVCV | 80.58 | 1071 | 0 | 798 |
| *Solanum juglandifolium* | TVCV | 83.07 | 2316 | 0 | 2045 |
| *Solanum mammosum* | TVCV | 80.77 | 2111 | 0 | 1613 |
| *Solanum marginatum* | TVCV | 81.26 | 1350 | 0 | 1062 |
| *Solanum seaforthianum* | TVCV | 83.49 | 1847 | 0 | 1692 |
| *Solanum stellatiglandulosum* | TVCV | 85.67 | 342 | 8.55E-97 | 359 |
| *Witheringia solanacea* | TVCV | 81.40 | 613 | 2.4E-136 | 492 |

**Online Resource 3.** RT-PCR analysis showing detection of potyvirus and CDV in *B. × candida* cultivars and sap-inoculated solanaceae species**.**

**
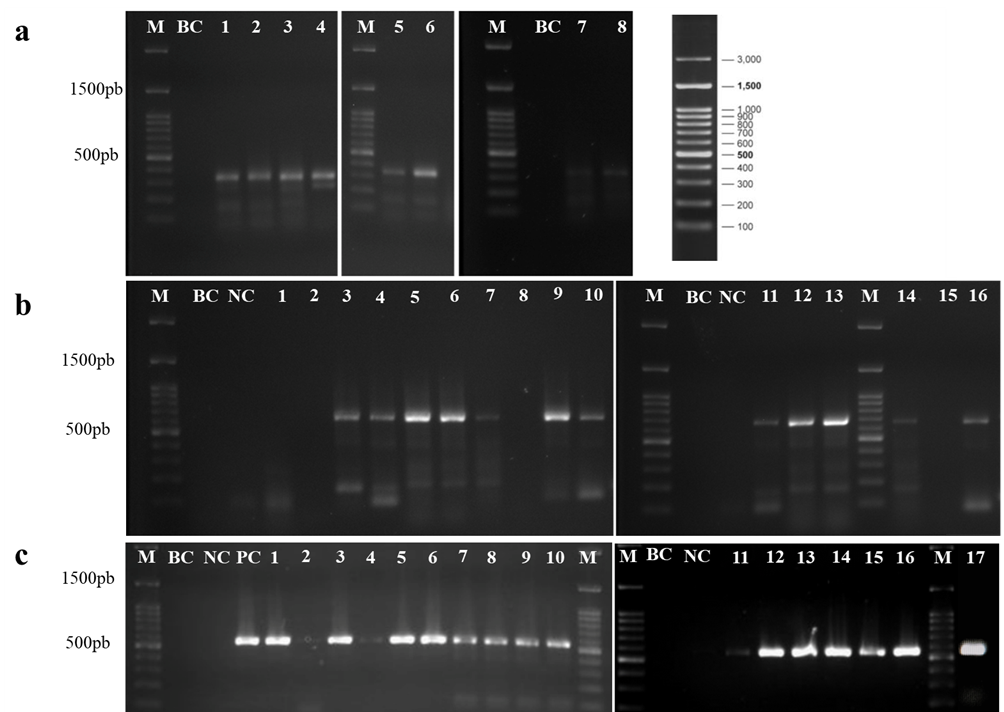
**

Abbreviations: **M,** molecular marker (100 bp + 3 kbp); **BC,** blank control; **NC,** negative control; **PC,** positive control; **DL,** deformed leaf; **OL,** ovate leaf; **SL,** slender leaf. Some lanes showed no amplification, possibly due to reaction inhibition or low template concentration.

**(a)** **RT-PCR amplification using degenerate primers targeting the NIb regions of potyviruses**. Lanes: 1, ‘Buyés 1’ (OL); 2, ‘Buyés 2’ (OL); 3, ‘Munchiro’ (DL); 4, ‘Dientes’ (OL); 5, ‘Ocre’ (OL); 6, ‘Amarón’ (DL); 7, ‘Quinde’ (OL); 8, ‘Quinde’ (DL). **(b) RT-PCR amplification using degenerate primers targeting the CI regions of potyviruses**. Lanes: 1 ‘Buyés 1’ (non PCR-product was observed); 2, ‘Búyes 2’ (non PCR-product was observed) ;3, ‘Amarón’ (OL); 4, ‘Amarón’ (DL); 5, ‘Quinde’ (OL); 6, ‘Quinde’ (DL); 7, ‘Ocre’ (OL); 8, ‘Culebro’ (non PCR-product was observed); 9–10, ‘Munchiro’ (DL); 11, ‘Buyés 1’ (OL); 12, ‘Buyés 2’ (OL); 13, ‘Munchiro’ (DL); 14, ‘Dientes’ (OL); 15, ‘Ocre’ (non PCR-product was observed); 16, ‘Culebro’ (SL). **(c)** **CDV detection using specific primers for the partial NIb/CP region, in *Brugmansia* cultivars and solanaceous plants inoculated with sap from *B. candida* cultivar ‘Munchiro’**. Lanes: 1, ‘Dientes’ (OL); 2, ‘Ocre’ (OL); 3, ‘Amarón’ (DL); 4, ‘Amarón’ (OL); 5, ‘Buyés 1’ (OL); 6, ‘Buyés 2’ (OL); 7, ‘Quinde’ (DL); 8, ‘Quinde’ (OL); 9, ‘Munchiro’ (DL); 10, ‘Culebro’ (SL); 11, *N. tabacum*; 12, *N. glutinosa*; 13, *B. sanguinea*; 14, *S. melongena*; 15, *P. peruviana*; 16, *P. hybrida*; 17, *S. quitoense*.

**Online Resource 4.** BLAST Analysis of partial CP gene sequences from CDV isolates infecting *Brugmansia* spp. cultivars and experimentally inoculated Solanaceae species

| **Sample** | **Pident %** | **evalue** | **Bitscore** |
| --- | --- | --- | --- |
| *B. × candida* cv. ‘Buyés 1’ | 99.16 | 4e-180 | 643 |
| *B. × candida* cv. ‘Quinde’ (irregular shape) | 98.32 | 4e-175 | 627 |
| *B. × candida* cv. ‘Amarón’ (irregular shape) | 99.43 | 9e-177 | 632 |
| *B. × candida* cv. ‘Amarón’ (Positive control) | 99.14 | 4e-175 | 627 |
| *B. × candida* cv. ‘Amarón’ (regular shape) | 93.84 | 2.16e-148 | 538 |
| *B. × candida* cv. ‘Buyés 2’ | 99.16 | 4e-180 | 643 |
| *B. × candida* cv. ‘Culebro’ | 99.44 | 0,0 | 649 |
| *B. × candida* cv. ‘Dientes’ | 99.16 | 4e-180 | 643 |
| *B. × candida* cv. ‘Munchiro’ | 99.44 | 0,0 | 649 |
| *B. × candida* cv. ‘Ocre’ | 98.88 | 2e-278 | 638 |
| *B. × candida* cv. ‘Quinde’ (regular shape) | 99.16 | 4e-180 | 643 |
| *B. sanguinea* | 99.44 | 0,0 | 649 |
| *N. glutinosa* | 97.49 | 4e-170 | 610 |
| *N. Tabacum* | 99.16 | 4e-180 | 643 |
| *Petunia hybrida* | 99.43 | 9e-177 | 632 |
| *Physalis peruviana* | 98.88 | 2e-178 | 638 |
| *Solanum melongena* | 99.44 | 0,0 | 649 |
